# Micro-Mechanism of Interfacial Separation and Slippage of Graphene/Aluminum Nanolaminated Composites

**DOI:** 10.3390/nano8121046

**Published:** 2018-12-13

**Authors:** Jia-Qi Zhu, Qing-Sheng Yang, Xiao-Qiao He, Kun-Kun Fu

**Affiliations:** 1 Department of Engineering Mechanics, Beijing University of Technology, Beijing 100124, China; zjq_006@emails.bjut.edu.cn; 2 Department of Civil and Architecture Engineering, City University of Hong Kong, Tat Chee Avenue, Kowloon, Hong Kong; 3 School of Aerospace Engineering and Applied Mechanics, Tongji University, Shanghai 200092, China; stone_fkk@163.com

**Keywords:** graphene/aluminum nanolaminated composite, interfacial separation, interfacial slippage, molecular dynamics, cohesive parameters

## Abstract

Due to their excellent properties and two-dimensional geometry, graphenes (Grs) have been widely used as reinforced fillers in graphene/aluminum nanolaminated composite (GANC). The separation and slippage behavior of the GANC is highly dependent on the interfacial properties between Gr and aluminum (Al). In this study, two interfacial failures of GANCs, i.e., pull-up failure and pull-out failure, were investigated using a molecular dynamics (MD) method. The effects of the crystal orientation of single-crystal Al component and the geometry of the Gr component on the normal and shear interfacial properties of the GANC were examined. It was evident that the interfacial pull-up resistance resulted from the atomic forces of all the atoms at the interface, whereas the interfacial shear force during pull-out stems from the atomic forces of the atoms at the crack tip. In addition, the studies revealed that the interface bonding strength between the Gr and Al was sensitive to both the crystal orientation of the Al and the environmental temperature. Finally, the cohesive law was used to describe the interfacial behavior of the Gr and Al, providing the interfacial data for the finite element modeling of composites with Gr and Al interface.

## 1. Introduction

Metal matrix composites (MMCs) reinforced by some carbon nanomaterials, such as carbon nanotube (CNT), graphene (Gr) and their derivatives [1,2,3,4,5], have been extensively used in applications such as aerospace and electronic packaging. The interface between the carbon filler and the matrix plays an important role in the overall mechanical properties of the MMCs because of the effective stress transfer [6]. Therefore, it is of importance to study the interfacial behavior of carbon filler and metal matrix.

Over the past few years, the shear interfacial properties between carbon nanomaterial (e.g., CNT) and metal or polymer matrix have been widely examined by pull-out loading [7,8,9,10,11,12]. For instance, the effects of the length, diameter and wall number of CNTs on the shear interfacial property between CNT and PMMA (polymethyl methacrylate) were studied using molecular dynamics (MD) simulations by Li et al. [13]. Experiments and MD simulations of CNT pull-out in a palladium matrix were conducted by Hartmann et al. [14,15], revealing that pull-out force is dependent on the diameter of the CNT and the defect inside the CNT. Recently, aluminum (Al) has gained more attention due to its lightweight nature [16]. However, the strength of Al is not promising, and therefore, the Al has increasingly been strengthened by carbon nanomaterials, making it important to investigate the interface properties between the carbon reinforcement and the Al matrix. For example, Silvestre et al. [17] investigated the reinforced mechanism of a CNT on the compressive properties of Al using an MD method. It was found that the reinforcement of the CNT on the Al can be attributed to both the intrinsic strength of the CNT and the interfacial sliding force between the CNT and Al. Song et al. [18] showed that the presence of nickel between CNT and Al increased the interfacial bonding strength. Moreover, a CNT pull-out process in an Al matrix was simulated by Liu et al. [19], and the pull-out force was correspondingly determined.

Since single layers of graphene (Gr) were first produced by Novoselov et al. [20], Gr has been considered as a promising reinforcing filler in MMCs because of its extraordinary mechanical properties, with up to 130 GPa strength and 1 TPa modulus [21,22,23,24,25]. As a single-atomic-layer material [26,27,28], the intrinsic two-dimensional geometry of the Gr is propitious to enhance the interface between metal matrix and the Gr compared to CNT. Shin et al. [29] showed that the mechanical properties of Gr reinforced MMCs was much higher than those of the MMCs with the addition of CNTs. With the successful synthesis of Gr/metal laminates, the interfacial properties of the Gr and metal have attracted more attention. Duan et al. [30] studied the interfacial properties between Gr and copper using an MD method and proposed a formula for predicting the interfacial strength. Chen et al. [31] studied the friction between Gr and three different substrates (i.e., Al, polymer and cementitious material) and adhesion at the crack surface using an MD method. The results showed that the interfacial shear strength increased with the increase of the crack expansion velocity, and the adhesion force on the crack tip provided a stable resistance during a Gr pull-out process. Shi et al. [32] studied the binding, exfoliation and folding behaviors of Gr on single-crystal coppers with different orientations. The results showed that the interfacial binding energy of Cu (111) is the highest compared with the Cu (001) and Cu (110). In recent years, Chu et al. [33] investigated the anisotropic mechanical properties of Gr/copper MMCs. It was seen that the strength of the copper was significantly improved with the addition of Gr parallel to the loading direction, whereas the strength decreased when the graphene was placed perpendicular to the direction of loading. Correspondingly, two distinct failure patterns at the interface of the MMCs was seen; that is, graphene slippage and graphene peer-off. The shear interfacial properties between Gr and Al were examined by Jiang et al. [34] based on a cohesive zone model using an MD method. Although extensive studies have been presented focusing on the Gr/metal MMCs using experimental method and MD simulation, the normal interfacial behavior of the Gr/metal MMCs is still unclear, especially for lightweight graphene/Al nanolaminated composite (GANC).

In this study, the interfacial separation and slippage behavior between Gr and Al in GANCs were investigated using an MD method in an effort to provide useful insights into the binding ability between Gr and Al and the mechanism of load transfer during failure process. The effects of the crystal orientation of the Al, the temperature and the size of the Gr on the interfacial behavior of the GANCs were examined. The normal and shear interfacial properties were investigated using pull-out and pull-up loadings, respectively. Finally, the cohesive law was used to describe the interfacial behavior of Gr and Al, providing the intrinsic material properties for the future finite element modeling. The present work, based on a simplified model, is of great importance to the further research in modeling and exploring the realistic interfacial issue of the GANC.

## 2. Model and Methodology

### 2.1. MD Models

**Figure 1:**
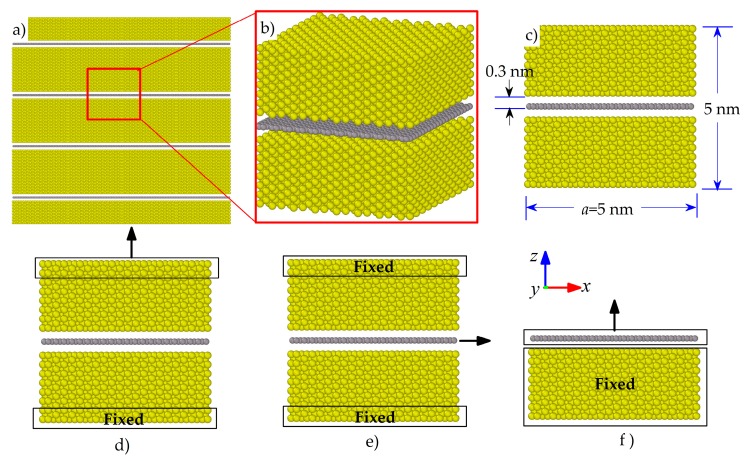
Schematic models of the GANC: (**a**) an overall model; (**b**) a RVE model; (**c**) the dimension of the RVE model; (**d**) pull-up simulation; (**e**) pull-out simulation; (**f**) cohesive law simulation.

A schematic of an overall GANC model with perfect interfaces is illustrated in Figure 1a. In the model, the Al atoms are arranged as a face-centered cubic (FCC) structure and the Gr layer is free from defects. A representative volume element (RVE) model was taken from the GANC to setup an MD model, as shown in Figure 1b. The model had dimensions of approximately 5 × 5 × 5 nm^3^, consisting of 8000 Al atoms and 1008 Gr atoms. The interlayer spacing between the Gr layer and Al was denoted as *d*, and *d* has a value of 3.0135 Å, as shown in Figure 1c. Figure 1d–f shows the three MD simulation models with different loading scenarios, i.e., pull-up simulation, pull-out simulation and cohesive law simulation, respectively. In addition, the pure Al models were established and the dimensions were approximately 5 × 5 × 5 nm^3^.

### 2.2. MD Potentials

Here, the interactions between Al-Al atoms were modelled using the embedded atom model (EAM) potential [35], and the adaptive intermolecular reactive empirical bond order (AIREBO) potential [36] was used to describe the interaction of Gr atoms. Meanwhile, because the interfacial interaction between graphene and aluminum mainly considers van der Waals interactions, the 12-6 Lennard-Jones (LJ) potential [17] well defined the weak interactions for carbon-metal systems. The function of these potentials is briefly introduced as follows.

In terms of the EAM potential, the total energy *E_i_* was given by

(1) $$E_{i}=F_{\alpha }\left(\underset{j\neq i}{\sum }\rho \beta \left(r_{ij}\right)+\frac{1}{2}\underset{j\neq i}{\sum }\emptyset \alpha \beta \left(r_{ij}\right)\right)$$

where *F* is the embedding energy, which is a function of the atomic electron density ρ. ∅ is the pair potential interaction, and α and β are the element types of atoms *i* and *j*, respectively. The multi-body nature of the EAM potential is a result of the embedding energy term. Both summations in the formula are over all neighbors *j* of atom *i* within the cutoff distance.

For the AIREBO potential, the total energy consists of three terms:

(2) $$E=\frac{1}{2}\underset{i}{\sum }\underset{j\neq i}{\sum }\left[E_{ij}^{REBO}+E_{ij}^{LJ}+\underset{k\neq i,j}{\sum }\underset{l\neq i,j,k}{\sum }E_{kijl}^{TORSION}\right]$$

The standard 6-12 Lennard-Jones potential was used to compute the interactions between Al and carbon atoms, which was given by

(3) $$E=4\varepsilon \left[{\left(\frac{\sigma }{r}\right)}^{12}-{\left(\frac{\sigma }{r}\right)}^{6}\right]\;\;\;\;r<r_{c}$$

where *ε* = 0.03457 eV, *σ* = 3.0135 Å and the cutoff distance $r_{c}$ is 8.5 Å.

### 2.3. MD Simulation Method

The MD simulations were carried out using an open-source MD program LAMMPS (Large-scale Atomic/Molecular Massively Parallel Simulator). The equilibrium state of the model must be reached before the MD simulation. The interfacial performances of Gr and Al with three different crystal surfaces (i.e., Al (001), Al (110) and Al (111)) were studied in this work. First, the whole system was subjected to an energy minimization process with a specified energy and a force tolerance of 1 × 10^−12^ by iteratively adjusting the atom coordinates. The timestep of one integration was set to 0.001 ps. Then, a relaxation process of 40 ps was performed at a constant temperature using an NVT ensemble where the number of molecules (N), volume (V), and temperature (T) remain constant. The temperature during this simulation was set to 1 K using a Nose-Hoover thermostat. When exploring the temperature effect, the same simulations were conducted under different temperatures (200 K, 300 K, 400 K, 500 K). Then, the structure of the MD model was optimized when the potential energy of the system was converged and an equilibrium state was achieved. After that, the settings of the three MD simulations (i.e., pull-up simulation, pull-out simulation, and cohesive law simulation) were detailed as follows.

**Pull-up simulation:** Pull-up simulations were performed to investigate the normal interfacial properties between Gr and Al in the direction perpendicular to the interface. Reaction force and displacement at the loading end were measured to examine the normal interfacial behavior of the composite. The interface may separate when a normal load was applied in the system, as shown in Figure 1d. Periodic boundary conditions were applied to the *x* and *y* directions and the free boundary condition was imposed to the loading direction (*z* direction). Three layers of Al atoms at the upper and lower ends of the MD model were fixed. Then, a constant displacement along the *z* direction was applied to the upper three layers atoms at a speed of 0.1 Å/ps under the NVT ensemble until the failure of the interface occurred. Correspondingly, the critical pull-up force was measured to determine the tensile performance and the normal interfacial strength of the GANC.

**Pull-out simulation:** Pull-out simulations were conducted to investigate the shear interfacial properties of the GANC. The Gr layer was pulled-out from the Al matrix under a pull-out force as shown in Figure 1e. Periodic boundary conditions were applied to the *y* and *z* directions and the free boundary condition was imposed to the loading direction (*x*-direction). After a same process of the energy minimization and equilibration of the whole system as used in the pull-up simulation, three layers of Al atoms at the upper and lower ends of the model were fixed. Then, a constant displacement along the *x*-direction at a speed of 0.1 Å/ps was applied to the three layers of atoms on the right end of Gr under the NVT ensemble until the Gr is completely pulled out from the Al matrix. The potential energy and pull-out force were recorded to investigate the shear interfacial behavior of the GANC.

**Cohesive law simulation:** A normal stress-separation relation (cohesive law) of the interface was obtained when the interface was subjected to a normal force, as shown in Figure 1f. After applying the same process of energy minimization and equilibration as was used in the pull-up simulation, all the Al atoms were fixed as a rigid body I and all the carbon atoms were fixed as a rigid body II. Then, a normal load that was the same as that used in the pull-up simulation was applied to the rigid body II until the Gr was completely detached from the Al layer, indicating that there was no interaction between the Gr atoms and Al atoms. After that, the potential energy and pull-up force were measured to investigate the cohesive behavior of the Gr and Al interface.

After the simulation, an open-source software Open Visualization Tool (OVITO) [37] was used to visualize the deformed structures.

## 3. Results and Discussion

### 3.1. Normal Interfacial Behavior by Pull-Up Simulation

**Figure 2:**
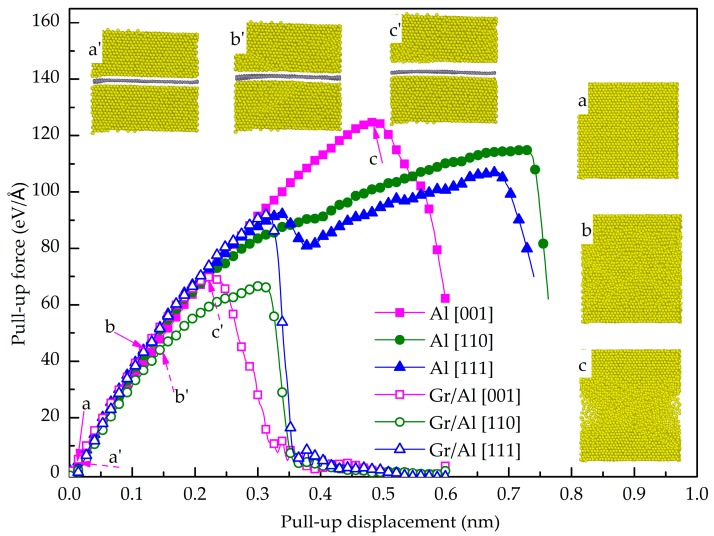
Pull-up force as a function displacement of the pure Al and GANC with three crystal orientations together with the structural deformations at different stages.

**Figure 3:**
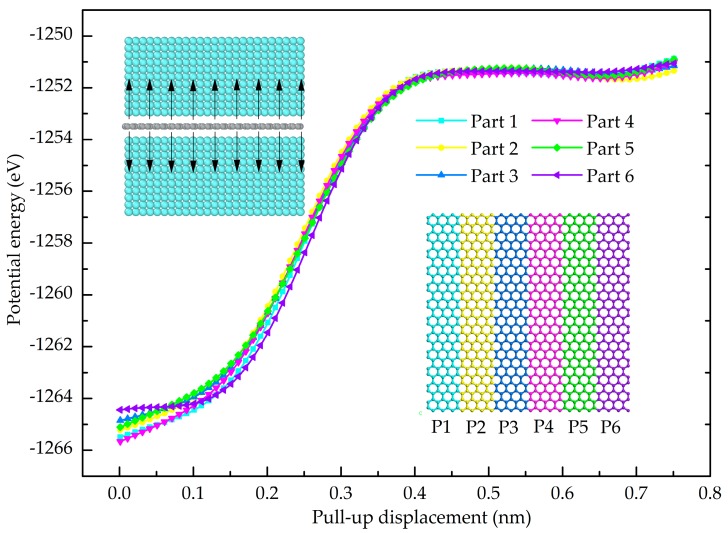
Variation of the potential energy of every part during pull-up process, the inserted figure is the segmentation of graphene sheet in pull-up test and the schematic diagram of load transfer at the interface.

Figure 2 shows the pull-up force as a function of displacement of the pure Al and the GANC models with three orientations of the Al, together with the corresponding structural deformations at different stages. It is seen that the lowest pull-up force of the pure Al models is 108.5 eV/A, higher than those of the GANCs. For example, the maximum pull-up force of the GANCs is 91.5 eV/A, occurring at the GANC with Al (111). The results in Figure 2 indicate that the existence of the Gr/Al interface degrades the tensile performance of the Al in the transverse direction, due to the fact that the interaction between Al and Gr atoms is weaker than that between Al and Al atoms. This finding is in line with the result from a previous experiment [33]. In addition, plastic deformation is detected in the pure Al model during the tension, as shown in Figure 2a–c. In contrast, the interface of the GANC fails prior to the occurrence of plastic deformation in the Al layer, as illustrated in Figure 2a’–c’. From the tensile force-displacement curves of the GANC and the corresponding microstructures of the composites in Figure 2, it is obvious that the force initially increases linearly with the increase of displacement, then decreases with the crack expansion. Finally, the force decreases to zero when the interface completely fails. It is seen from the microstructure of the nanolaminate that the separation of the composite material interface leads to the rapid decrease in the force, indicating the interface fracture failure. Therefore, the peak force of the force-displacement curve is selected to characterize the maximum bearing capacity of the interface. The normal strength of the interface is approximately equal to the tensile failure stress of the composite, which is calculated by dividing the applied maximum pull-up force by the cross-sectional area:

(4) $$\sigma =\frac{F}{A}=\frac{F}{S}=\frac{F_{\mathrm{pull}-\mathrm{up}\;\max}}{\mathrm{wL}}$$

where *w* and *L* represent the width and length of the graphene sheet, respectively. *A* denotes the interfacial contact area between the graphene and Al. *S* indicates the area of two-dimensional graphene sheets (*S* = *wL*). Using Equation (4), the normal strengths of the interface between the Gr and Al (001), Al (110) and Al (111) are 4.72 GPa, 4.08 GPa and 5.63 GPa, respectively. The Gr and Al (111) has the greatest normal interfacial strength. This finding is consistent with the conclusion obtained by a simulation on Gr/Cu nanolaminates [32]. To examine the contribution of atoms on the interfacial behavior, the Gr layer is divided into six equal parts (each part has the same number of atoms) as shown in the insert of Figure 3. Figure 3 shows the potential energy as a function of the pull-up displacement for each part. It is evident that the potential energy curves of the six parts coincide with each other, indicating that all the Al atoms at the interface make the equal contribution to interact with the C atoms during the pull-up. In addition, the Al (111) is the dense structure of an FCC metal and it has the largest number of Al-C interaction. Consequently, the Al (111) and Gr has the strongest interface bonding strength.

**Figure 4:**
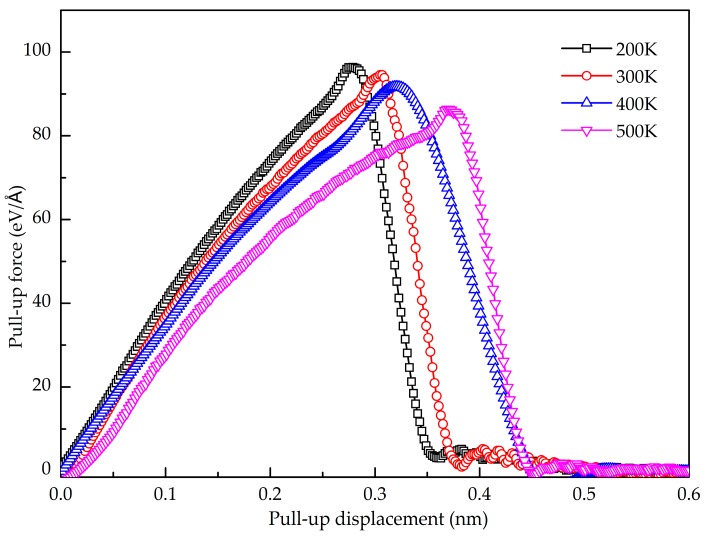
Pull-up force-displacement curve of the GANC at different temperatures.

**Figure 5:**
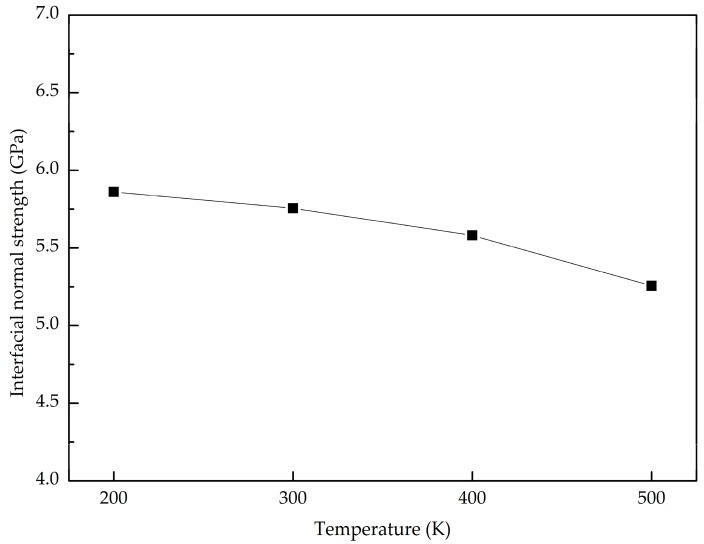
Normal interfacial strength of the GANC at different temperatures.

Next, we examine the temperature effect on the normal interfacial properties of the GANCs with Al (111). Figure 4 shows the pull-up force-displacement curves at temperatures ranging from 200 K to 500 K. Under the same displacement, the pull-up force is higher at a lower temperature. Figure 5 summarizes the normal interfacial strength at different temperatures. It is obvious that the normal interfacial strength decreases slightly with the increase of the temperature. At the atomic scale, atoms in crystals vibrate naturally around their equilibrium lattice positions due to the thermal fluctuation [38]. The higher temperature induces the more violent vibration. In the pull-up process, the increase in the temperature leads to the increase of the atomic velocity vibration. Therefore, the interface failure is easy to achieve at a high temperature.

**Figure 6:**
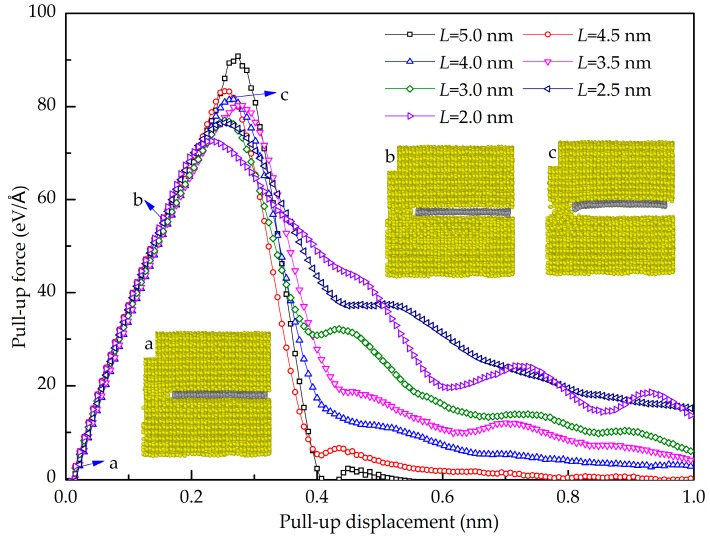
Pull-up force-displacement curves for the Gr/Al composites with different embedded length of graphene and the structural deformation diagram at different stage.

Here, the embedded length of Gr in Al is considered. Figure 6 shows the pull-up force-displacement curves of the GANC with different embedded lengths *L* of the Gr together with the corresponding structural deformation at different stages. The initial configuration of the model is shown in Figure 6a. As the load increases, the model in the *z*-direction is elongated and the distance of the Al-C atoms at the interface is slightly increased as shown in Figure 6b. When the pull-up force reaches its peak, the interface between the graphene and the Al is separated as shown in Figure 6c, resulting in the sudden drop in the force. It is seen that the maximum pull-up force increases with the increase of the embedment length of the Gr. It can be seen from Figure 6 that the sudden drop of the force-displacement curves is caused by the failure of the interface. After the peak, only the plastic deformation occurs in the Al atoms and is not discussed here.

### 3.2. Shear Interfacial Properties by Pull-Out Simulations

**Figure 7:**
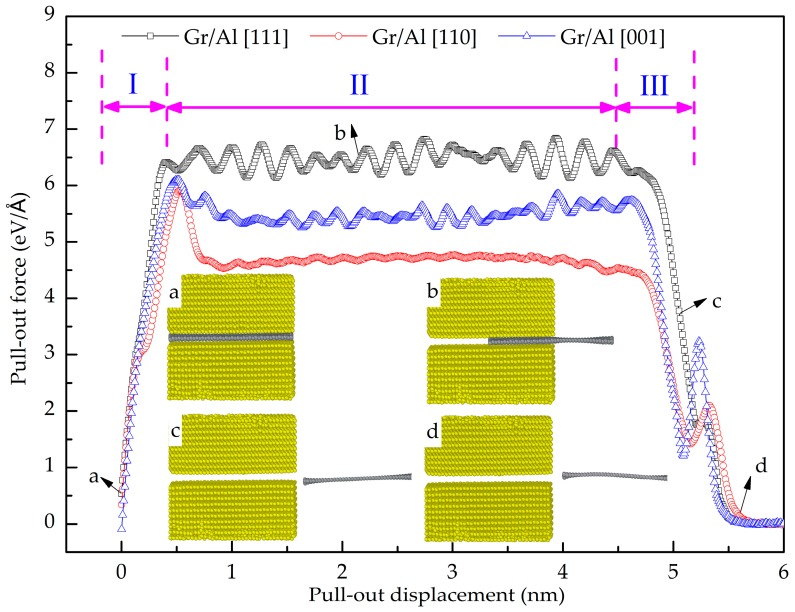
Pull-out force as a function of displacement with different Al crystal orientations.

**Figure 8:**
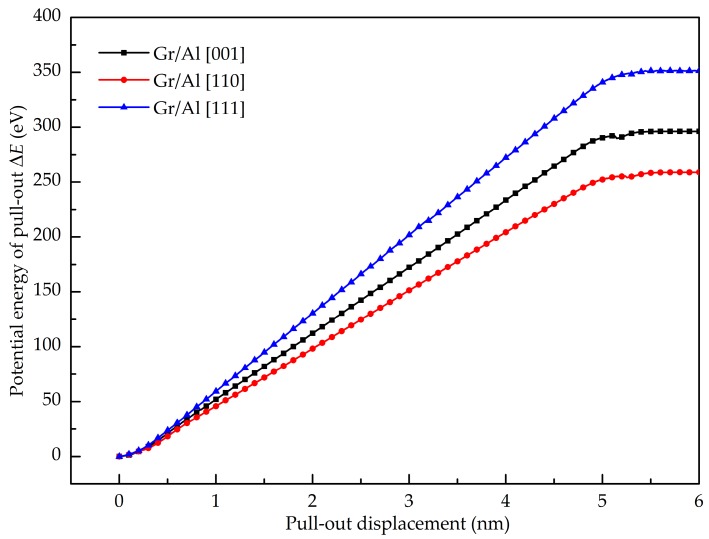
Potential energy increment of the different Al crystal orientations during pull-out test.

**Figure 9:**
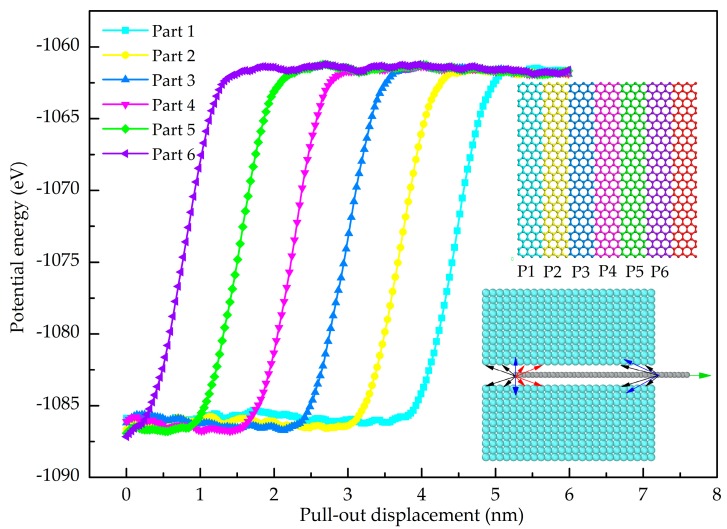
Variation of the potential energy of every part during pull-up process, the inset figure is the segmentation of graphene sheet at the non-loaded end (except for the red part) in pull-up test and the schematic diagram of load transfer at the interface.

In this section, the shear interfacial behavior of the Gr and Al is studied using a pull-out simulation. Figure 7 shows the pull-out force as a function of pull-out displacement of the GANCs with different Al orientations. It is evident that the Gr pull-out process can be divided into three stages, i.e., stage I, II and III. In stage I, the Gr is embedded in the Al, as shown in Figure 7a. Subsequently, the pull-out force gradually increases and reaches the stage II where the pull-out force is stable with increasing the displacement as shown in Figure 7b. Finally, the pull-out force drops with the Gr detaching from the Al, and the distance between Al and C atoms is less than the cut-off radius of Van der Waals interactions, as shown in Figure 7c. The pull-out force drops to zero, implying that the distance between graphene and Al beyond the cut-off radius of Van der Waals interactions, as shown in Figure 7d. In addition, it can be seen from the curves that the pull-out force of Gr/Al (111) nanolaminate is highest in stage II. Correspondingly, the shear interfacial strength of the GANCs can be calculated. The interfacial shear strength is defined as the ratio of the maximum pull-out force and contact area. However, due to the fluctuation of the forces in stage II, the average force in stage II is selected to calculate the shear interfacial strength [39]. In the pull-out process, the increase of energy is equivalent to the work done by the pull-out force. Therefore, we have

(5) $$E_{pull-out}=\int_{x=0}^{x=L}A\tau_{i}dx=\int_{x=0}^{x=L}2S\tau_{i}dx=w\tau_{i}L^{2}$$

with

(6) $$\tau_{i}=\frac{E_{pull-out}}{wL^{2}}$$

and then

(7) $$E_{pull-out}=E_{pe2}-E_{pe1}$$

where *E*_pull-out_ is the potential energy increments of the pull-out processes. The interfacial shear strength is expressed by *τ*_i_. *E*_pe1_ and *E*_pe2_ indicate the potential energy of composites before the loading and after the separation of the Gr and Al, respectively. The potential energy increment of pull-out is shown in Figure 8. The interfacial shear strength between the Gr and Al (001), Al (110) and Al (111) is found as 296 MPa, 258 MPa and 351 MPa, respectively. The Gr and Al (111) exhibits the highest interfacial shear strength. To examine the contribution of atoms on the shear interfacial behavior, the Gr (in addition to the red part) is divided into six equal parts (see the inset figure of Figure 9). Figure 9 shows the potential energy as a function of pull-out displacement of the six parts. It is obviously seen that the potential energy of the sixth part (P6: purple) increase first during the pull-out process, then the other parts in turn. Hence, it is concluded that the maximum pull-out force is related to the number of atoms at the crack tip. Furthermore, the number of atoms at the crack tip represents the width of the graphene sheet; it was concluded by Duan et al. [30] that the pull-out force is related to the width of the graphene sheet, but has nothing to do with the length. In the present work, a reasonable explanation is given to prove the interesting phenomenon through the curves of potential energy. In the pull-out process as shown in the inset of Figure 9, the black arrows denote the attractive force, the red arrows represent the repulsive force, and the blue arrows represent the resultant force. Because the C atoms in the Gr are subjected to both the attraction and repulsion forces of the adjacent Al atoms, the resultant force of attraction and repulsion is almost balanced out due to the symmetry. Therefore, the C atoms inside the Al matrix have no contribution on the generated pull-out force. In contrast, the atoms at the crack tip (the right end) contribute to the pull-out force due to the attractions of the Al atoms.

**Figure 10:**
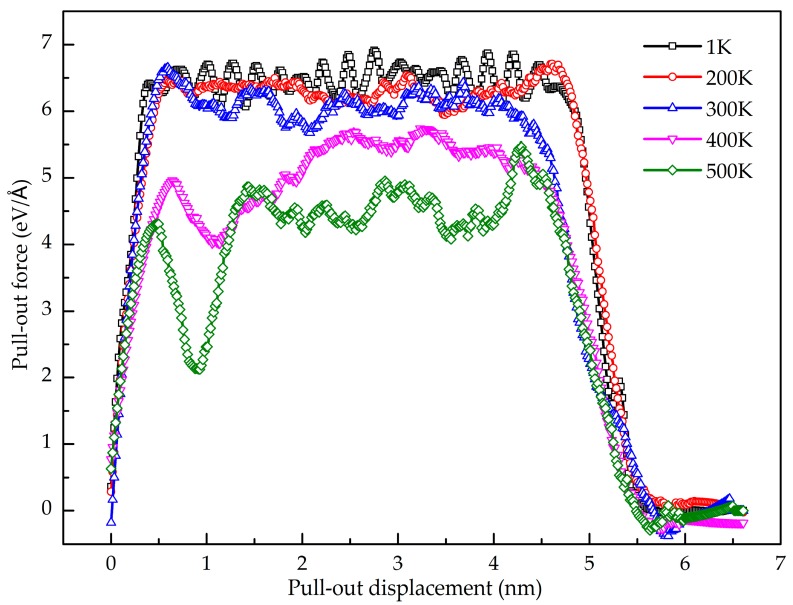
Pull-out force as a function of displacement at different temperature.

**Figure 11:**
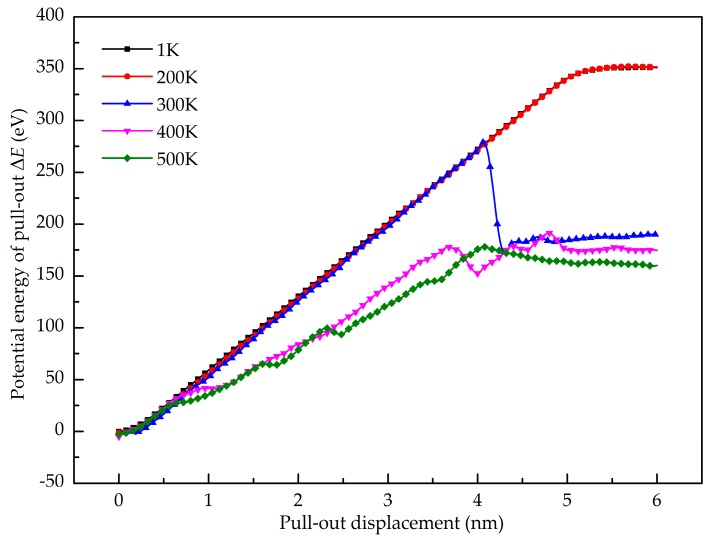
Potential energy increment during pull-out test under different temperature.

**Figure 12:**
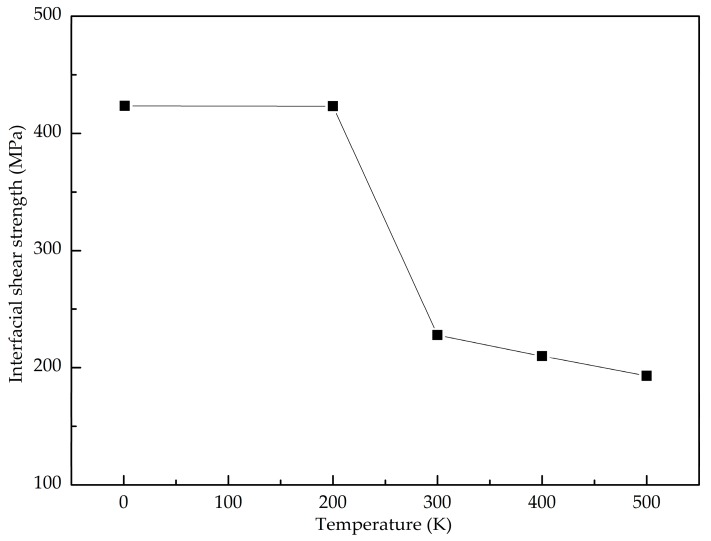
Interfacial shear strength under different temperatures.

The temperature effect on the shear interfacial behavior is investigated here. Figure 10 shows the pull-out force as a function of pull-out displacement at different temperatures. It is found that the value of the stable pull-out force decreases with the increase of the temperature from 200 K to 500 K. When the temperature is lower than 200 K, the peak pull-out force varies little. Figure 11 shows the potential energy increment during the pull-out simulation at different temperatures, and the calculated interfacial shear strength is shown in Figure 12. It is seen that the shear interfacial strength keeps unchanged below 200 K with a value of 423 MPa. As the temperature rises from 200 K, the interfacial shear strength decreases. At the elevated temperature above 300 K, a slight drop of the interfacial shear strength is detected. At higher temperatures, the GANC becomes softer with lower strength and modulus. The values of pull-out force and interfacial shear strength at high temperatures are lower than those at low temperatures, due to the thermal fluctuation.

**Figure 13:**
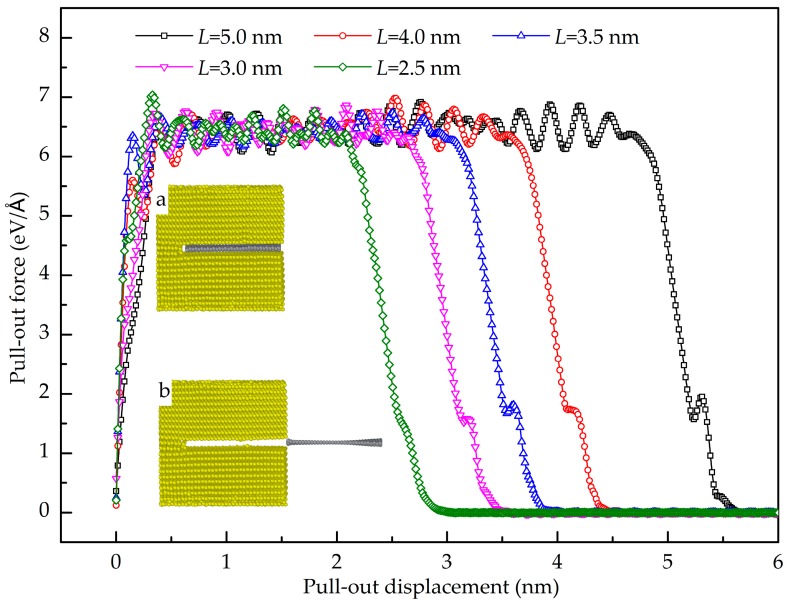
Pull-out force-displacement curves for the Gr/Al composites with different embedded length of graphene and the structural deformation diagram at different stage.

**Figure 14:**
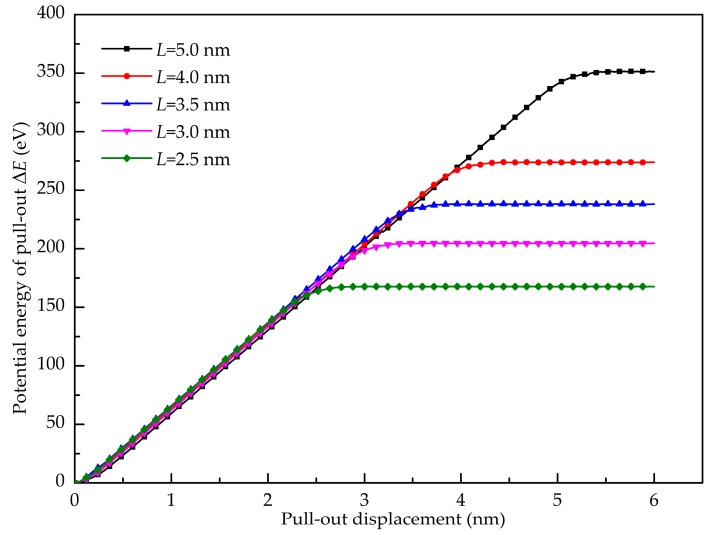
Potential energy increment during pull-out test with different embedded length of graphene.

**Figure 15:**
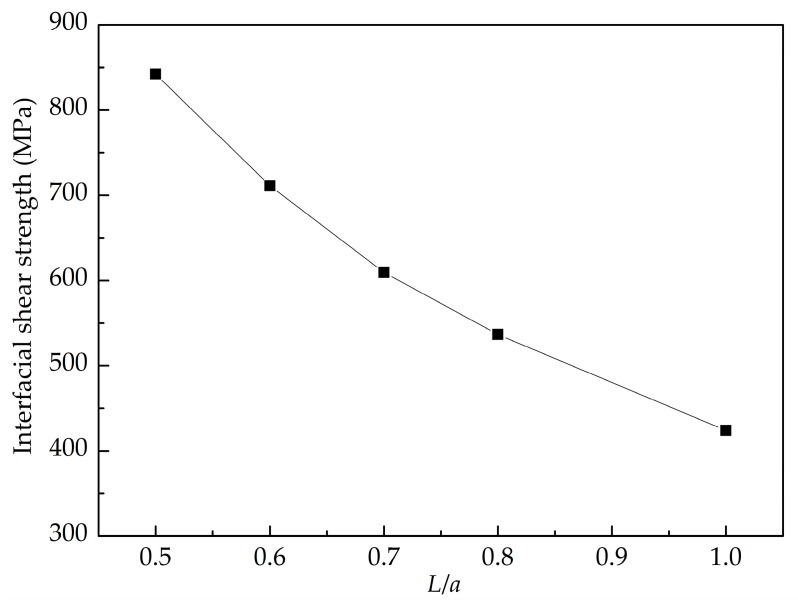
Interfacial shear strength of the composite with different embedded length of graphene.

Figure 13 shows the pull-out force-displacement curves of the GANC with different embedded lengths of the Gr and the corresponding structural deformation at different stages. It is obvious that the maximum pull-out force is insensitive to the embedded length of the Gr. As already mentioned, the maximum pull-out force is dependent on the attraction force of atoms at the crack tip. The embedded length of the Gr does not change the number of atoms at the crack tip. Hence, the maximum pull-out force remains constant with different embedded length. Figure 14 presents the potential energy increment during the pull-out simulations with different embedded lengths of the graphene. When *L* is larger, the work done by the pull-out force is greater, leading to a greater potential energy increment. The shear interfacial strength of the composites with different embedded lengths of the graphene are calculated as shown in Figure 15. It can be clearly seen that the shear interfacial strength is controlled by the embedded length of the Gr, and the interfacial shear strength is increased due to the decreased dimension of the Gr.

### 3.3. Cohesion Law of Gr/Al Interface

**Figure 16:**
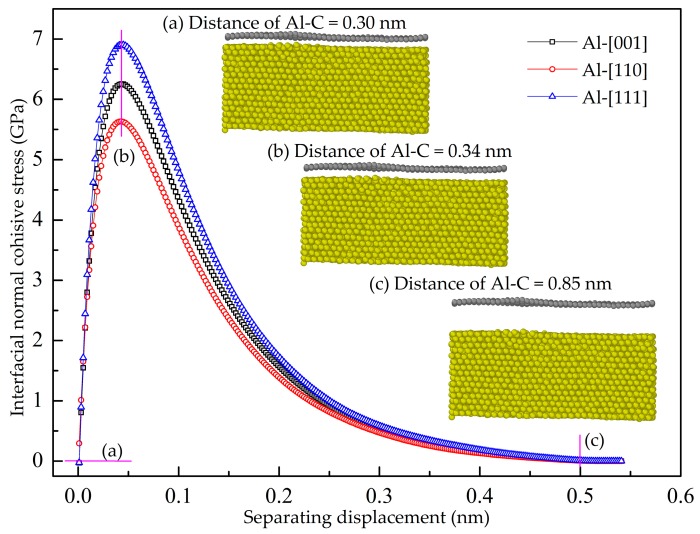
Interfacial normal cohesive stress—separating displacement curves.

**Figure 17:**
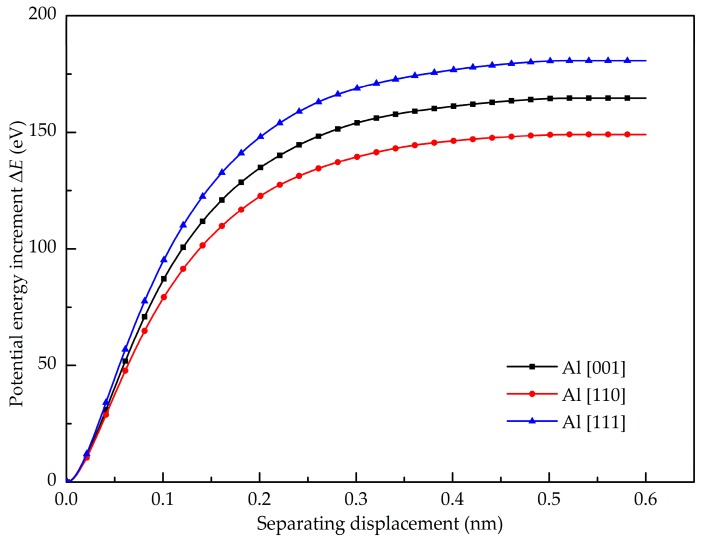
Potential energy increment during the cohesive law simulation.

In the cohesive law simulation, the Gr and Al are defined as rigids after the structure achieves the equilibrium state. Therefore, the plastic deformation inside the graphene and Al is neglected, and the measured force and displacement are caused due to the interaction between the C atoms and Al atoms in the interface. Figure 16 shows normal cohesive stress-displacement curves of the GANC with different Al orientations. The initial distance between the graphene and Al is 0.30135 nm, as seen in Figure 16a, which is due to the fact that the zero potential energy distance of LJ potential between Al-C is 0.30135 nm. When the loading distance is 0.04 nm, the cohesive stress-displacement curves reach the peak. At this stage, the cracking distance between the Gr and Al is 0.34 nm, as seen in Figure 16b. Finally, when the cohesive stress of the interface drops to zero and the interface is completely cracked, the distance between the graphene and Al is about 0.85 nm, as shown in Figure 16c. When the cracking distance of the interface reaches the cutoff radius of the Van der Waals force (0.85 nm), there is no interaction between the Gr and Al, implying that the interface is completely fractured. It is evident that the interfacial normal cohesive stress of the interface between the Gr and Al (111) is the highest. The curves can be divided into two stages [40]. First, the cohesion of the interface rises rapidly and reaches its peak with the increase of displacement. Second, the cohesive stress declines slowly with the increase of displacement. The interfacial normal cohesive strength of the composites with (001)-stacking Al, (110)-stacking Al and (111)-stacking Al is 6.25 GPa, 5.63 GPa and 6.91 GPa, respectively, corresponding to the three highest stresses of the three curves. The area of cohesive stress-displacement curve is 0.912 J/m^2^, 0.820 J/m^2^ and 1.010 J/m^2^, respectively [41], representing the interfacial fracture energy or cohesive energy. Figure 17 shows the potential energy increment during the cohesive law simulation. It is evident that the Gr separated from the Al (111) surface releases the highest energy which indicates that a higher critical load is needed to break the interface of the Gr and Al (111). The present work obtains the cohesive strength and cohesive energy of the interface between the Gr and Al, which can be further used for finite element modeling.

## 4. Conclusions

In this study, the micro-mechanisms of interfacial separation and slippage of the GANC were studied by the pull-up, pull-out and the cohesive law simulations via molecular dynamics method and revealed the mechanism of force transfer during interface failure. The following conclusions can be drawn:

1. The maximum pull-up force of the GANC is lower than that of pure Al, indicating that the presence of the Gr/Al interface reduces the tensile property of the Al. The GANC with the Al (111) exhibits the highest normal interfacial strength due to the dense arrangement of the Al atoms. In addition, the normal interfacial force decreases with the reduction of the embedded length of the graphene because all the atoms on the interface contribute to the pull-up force.
2. The GANC with the Al (111) exhibits the highest normal interfacial strength because the number of atoms at the creak tip is the most. The embedded length of the graphene does not affect the pull-out force, because only a column of atoms at the crack tip acts during the pull-out process, whereas the shear interfacial strength increases with the decrease of the embedded length of graphene. Furthermore, the interface of the GANC has a better resistance at a lower temperature.
3. The relation of normal stress and separating displacement of the interface was studied and a cohesive law for the finite element simulation of the interface was determined.

In the present work, we discussed two kinds of interfacial failure of GANC with ideal structure, while the inherent randomness of the problem due to different types of defects in the Gr and the interface are ignored which can be investigated in future work. It should be noted that the mechanical properties of the composite were underestimated because of the simplified model with ideal structure, which may be not obtained if more realistic model was used.

## References

[1] Moghadam A.D., Schultz B.F., Ferguson J.B., Omrani E., Rohatgi P.K., Gupta N. (2014). Functional metal matrix composites: Self-lubricating, self-healing, and nanocomposites-an outlook. JOM.

[2] Singh M., Rana R.S., Purohit R., Sahu K. (2015). Development and analysis of al-matrix nano composites fabricated by ultrasonic assisted squeeze casting process. Mater. Today Proc..

[3] Khalil I., Rahmati S., Julkapli N.M., Yehye W.A. (2018). Graphene metal nanocomposites—Recent progress in electrochemical biosensing applications. J. Ind. Eng. Chem..

[4] Kaczmar J.W., Pietrzak K., Osiński W. (2000). The production and application of metal matrix composite materials. J. Mater. Process. Technol..

[5] Rawal S.P. (2001). Metal-matrix composites for space applications. JOM.

[6] Kashfipour M.A., Mehra N., Zhu J. (2018). A review on the role of interface in mechanical, thermal, and electrical properties of polymer composites. Adv. Compos. Hybrid Mater..

[7] Awasthi A.P., Lagoudas D.C., Hammerand D.C. (2009). Modeling of graphene-polymer interfacial mechanical behavior using molecular dynamics. Model. Simul. Mater. Sci. Eng..

[8] Chen Y.L., Liu B., Huang Y., Hwang K.C. (2011). Fracture toughness of carbon nanotube-reinforced metal- and ceramic-matrix composites. J. Nanomater..

[9] Chowdhury S.C., Okabe T. (2007). Computer simulation of carbon nanotube pull-out from polymer by the molecular dynamics method. Compos. Part A-Appl. Sci. Manuf..

[10] Coto B., Antia I., Barriga J., Blanco M., Sarasua J. (2013). Influence of the geometrical properties of the carbon nanotubes on the interfacial behavior of epoxy/cnt composites: A molecular modelling approach. Comput. Mater. Sci..

[11] Peng X., Meguid S.A. (2017). Molecular simulations of the influence of defects and functionalization on the shear strength of carbon nanotube-epoxy polymer interfaces. Comput. Mater. Sci..

[12] Sindu B.S., Sasmal S. (2015). Evaluation of mechanical characteristics of nano modified epoxy based polymers using molecular dynamics. Comput. Mater. Sci..

[13] Li Y., Liu Y., Peng X., Yan C., Liu S., Hu N. (2011). Pull-out simulations on interfacial properties of carbon nanotube-reinforced polymer nanocomposites. Comput. Mater. Sci..

[14] Hartmann S., Blaudeck T., Hölck O., Hermann S., Schulz S.E., Gessner T., Wunderle B. (2014). Quantitativein-situ scanning electron microscope pull-out experiments and molecular dynamics simulations of carbon nanotubes embedded in palladium. J. Appl. Phys..

[15] Hartmann S., Wunderle B., Hölck O. (2012). Pull-out testing of swcnts simulated by molecular dynamics. Int. J. Theor. Appl. Nanotechnol..

[16] Bartolucci S.F., Paras J., Rafiee M.A., Rafiee J., Lee S., Kapoor D., Koratkar N. (2011). Graphene–aluminum nanocomposites. Mater. Sci. Eng. A.

[17] Silvestre N., Faria B., Canongia Lopes J.N. (2014). Compressive behavior of cnt-reinforced aluminum composites using molecular dynamics. Compos. Sci. Technol..

[18] Song H., Zha X. (2010). Influence of nickel coating on the interfacial bonding characteristics of carbon nanotube–aluminum composites. Comput. Mater. Sci..

[19] Liu S., Hu N., Yamamoto G., Cai Y., Zhang Y., Liu Y., Li Y., Hashida T., Fukunaga H. (2011). Investigation on cnt/alumina interface properties using molecular mechanics simulations. Carbon.

[20] Novoselov K.S., Geim A.K., Morozov S.V., Jiang D., Zhang Y., Dubonos S.V., Grigorieva I.V., Firsov A.A. (2004). Electric field effect in atomically thin carbon films. Science.

[21] Kim Y., Lee J., Yeom M.S., Shin J.W., Kim H., Cui Y., Kysar J.W., Hone J., Jung Y., Jeon S. (2013). Strengthening effect of single-atomic-layer graphene in metal–graphene nanolayered composites. Nat. Commun..

[22] King A., Johnson G., Engelberg D., Ludwig W., Marrow J. (2008). Observations of intergranular stress corrosion cracking in a grain-mapped polycrystal. Science.

[23] Lee C., Wei X., Kysar J.W., Hone J. (2008). Measurement of the elastic properties and intrinsic strength of monolayer graphene. Science.

[24] Wang L., Yang Z., Cui Y., Wei B., Xu S., Sheng J., Wang M., Zhu Y., Fei W. (2017). Graphene-copper composite with micro-layered grains and ultrahigh strength. Sci. Rep..

[25] Zhu Y., Murali S., Cai W., Li X., Suk J.W., Potts J.R., Ruoff R.S. (2010). Graphene and graphene oxide: Synthesis, properties, and applications. Adv. Mater..

[26] Mas-Balleste R., Gomez-Navarro C., Gomez-Herrero J., Zamora F. (2011). 2d materials: To graphene and beyond. Nanoscale.

[27] Lu Q., Huang R. (2009). Nonlinear mechanics of single-atomic-layer graphene sheets. Int. J. Appl. Mech..

[28] Cao M., Xiong D., Tan Z., Ji G., Amin-Ahmadi B., Guo Q., Fan G., Guo C., Li Z., Zhang D. (2017). Aligning graphene in bulk copper: Nacre-inspired nanolaminated architecture coupled with in-situ processing for enhanced mechanical properties and high electrical conductivity. Carbon.

[29] Shin S.E., Choi H.J., Shin J.H., Bae D.H. (2015). Strengthening behavior of few-layered graphene/aluminum composites. Carbon.

[30] Duan K., Li L., Hu Y., Wang X. (2017). Interface mechanical properties of graphene reinforced copper nanocomposites. Mater. Res. Express.

[31] Chen S.J., Li C.Y., Wang Q., Duan W.H. (2017). Reinforcing mechanism of graphene at atomic level: Friction, crack surface adhesion and 2d geometry. Carbon.

[32] Shi X., Yin Q., Wei Y. (2012). A theoretical analysis of the surface dependent binding, peeling and folding of graphene on single crystal copper. Carbon.

[33] Chu K., Wang F., Wang X., Huang D. (2018). Anisotropic mechanical properties of graphene/copper composites with aligned graphene. Mater. Sci. Eng. A.

[34] Jiang W., Wu Y., Qin Q., Li D., Liu X., Fu M. (2018). A molecular dynamics based cohesive zone model for predicting interfacial properties between graphene coating and aluminum. Comput. Mater. Sci..

[35] Daw M.S., Baskes M.I. (1984). Embedded-atom method—Derivation and application to impurities, surfaces, and other defects in metals. Phys. Rev. B.

[36] Brenner D.W., Shenderova O.A., Harrison J.A., Stuart S.J., Ni B., Sinnott S.B. (2002). A second-generation reactive empirical bond order (rebo) potential energy expression for hydrocarbons. J. Phys. Condens. Mattter.

[37] Stukowski A. (2010). Visualization and analysis of atomistic simulation data with ovito-the open visualization tool. Model. Simul. Mater. Sci. Eng..

[38] Duan K., Zhu F., Tang K., He L., Chen Y., Liu S. (2016). Effects of chirality and number of graphene layers on the mechanical properties of graphene-embedded copper nanocomposites. Comput. Mater. Sci..

[39] Lv C., Xue Q., Xia D., Ma M., Xie J., Chen H. (2010). Effect of chemisorption on the interfacial bonding characteristics of graphene−polymer composites. J. Phys. Chem. C.

[40] Jiang L., Tan H., Wu J., Huang Y., Hwang K. (2007). Continuum modeling of interfaces in polymer matrix composites reinforced by carbon nanotubes. Nano.

[41] Tan H., Jiang L.Y., Huang Y., Liu B., Hwang K.C. (2007). The effect of van der waals-based interface cohesive law on carbon nanotube-reinforced composite materials. Compos. Sci. Technol..

